# Identifying Cancer-Specific circRNA–RBP Binding Sites Based on Deep Learning

**DOI:** 10.3390/molecules24224035

**Published:** 2019-11-07

**Authors:** Zhengfeng Wang, Xiujuan Lei, Fang-Xiang Wu

**Affiliations:** 1 School of Computer Science, Shaanxi Normal University, Xi’an 710119, China; zfwang@snnu.edu.cn; 2 College of Information Science and Engineering, Guilin University of Technology, Guilin 541004, China; 3 Department of Mechanical Engineering and Division of Biomedical Engineering, University of Saskatchewan, Saskatoon, SK S7N 5A9, Canada; faw341@mail.usask.ca

**Keywords:** circRNA, RNA binding protein, cancer-specific, convolutional neural network

## Abstract

Circular RNAs (circRNAs) are extensively expressed in cells and tissues, and play crucial roles in human diseases and biological processes. Recent studies have reported that circRNAs could function as RNA binding protein (RBP) sponges, meanwhile RBPs can also be involved in back-splicing. The interaction with RBPs is also considered an important factor for investigating the function of circRNAs. Hence, it is necessary to understand the interaction mechanisms of circRNAs and RBPs, especially in human cancers. Here, we present a novel method based on deep learning to identify cancer-specific circRNA–RBP binding sites (CSCRSites), only using the nucleotide sequences as the input. In CSCRSites, an architecture with multiple convolution layers is utilized to detect the features of the raw circRNA sequence fragments, and further identify the binding sites through a fully connected layer with the softmax output. The experimental results show that CSCRSites outperform the conventional machine learning classifiers and some representative deep learning methods on the benchmark data. In addition, the features learnt by CSCRSites are converted to sequence motifs, some of which can match to human known RNA motifs involved in human diseases, especially cancer. Therefore, as a deep learning-based tool, CSCRSites could significantly contribute to the function analysis of cancer-associated circRNAs.

## 1. Introduction

Circular RNAs (circRNAs) are non-coding RNAs that have covalent and closed loop structures; thereby, they are more stable than most linear RNAs in cells [1]. Although circRNAs have been identified over twenty years, the biological functions of circRNAs remain largely unknown. For a long time, they have been generally thought to represent splicing errors and are expressed at low levels [2]. In recent years, an abundance and diversity of circRNAs were discovered in tissue and organ development by high-throughput sequencing [3,4], including many tissue-specific [5] and cell-specific [6] circRNAs, which may play a role in various human disorders and biological processes [7]. More specific functions were also discovered, such as regulating transcriptional initiation [8], affecting alternative splicing, and functioning as microRNA (miRNA) [9] or RNA binding protein (RBP) sponges [10,11].

In addition, several databases of circRNAs have been built for studying circRNAs. For instance, circBase collects and unifies data sets of circRNAs and provides scripts to identify circRNAs in sequencing data [12]. circRNADb provides detailed annotations of human circRNAs, including genomic information, exon splicing, genome sequence, internal ribosome entry site (IRES), open reading frame (ORF), and references [13]. CircR2Disease curates a database for experimentally supported associations between circRNAs and diseases, and provides a platform for investigating mechanism of the disease-related circRNAs [14]. CSCD (cancer-specific circRNAs database) is a database developed for cancer-specific circRNAs (CS-circRNAs), provides miRNA target sites, RBP binding sites, and potential open reading frames (ORFs) in CS-circRNAs [15].

With the deep understanding of circRNA regulatory functions, the control and function of circRNAs seem to largely rely on the specificity of RBPs. They participate in almost every phase of the circRNA life cycle, including formation [16], translation, post-transcriptional regulation [17], and extracellular transport [18]. In order to identify the interactions between RNAs and RBPs at the transcriptome-wide level, some CLIP (cross-linking and immunoprecipitation)-based experimental technologies in vivo were designed. Among them, RNAcompete provides binding affinities of specific RBPs to RNA probes [19]. Recently, CLIP-seq [20] has become the standard experimental procedure, with several variants including HITS-CLIP [20], PAR-CLIP [21], and iCLIP [22]. Additionally, it can also be applied to detect potential binding sites on unreported sequences.

Considering the cost-heavy and labor-intensive aspects of these biological experimental technologies, some computational methods [23,24], in particular, deep learning-based approaches, have been designed for identifying interactions between RNAs/DNAs and RBPs. Convolutional neural networks (CNNs) have been proven to be very successful in solving sequence-based problems [25,26]; they are usually employed to learn comprehensive features from the raw input data, and especially, the kernels in a CNN can be regarded as a motif scanner to detect the motifs in genomics. The recent application of CNN-based methods in genomics indicates its effectiveness in computational biology. For instance, DeepBind predicts the sequence specificities of DNA- and RNA-binding proteins based on CNNs [27], and is enhancing the prediction of sequence specificities of DNA binding proteins [28]. Zeng et al. present a CNN-based deep learning architecture for predicting DNA sequence binding sites using the ChIP-seq dataset [29]. iDeep [30], iDeepE [31], and iDeepS [32] were designed to identify RBP binding preferences on RNA sequences using CNNs and RNNs (recurrent neural networks). In addition, GraphProt is also a computational framework that can find RBP sequence- and structure-binding preferences from the high-throughput experimental data [33]. However, there is still no computational method for identifying the cancer-specific RBP binding sites on circRNAs.

In this study, we present a deep learning-based method to detect the cancer-specific RBP binding sites on circRNAs utilizing the RBP binding sites on CS-circRNAs data, which is driven by a concatenate convolutional neural network model. This method is compared with the conventional classifiers and other representative deep learning-based methods using sequences alone, and the results show its better prediction accuracy. In addition, the features learnt by CSCRSites are converted to sequence motifs and are compared with known human RNA motifs involved in human cancer diseases. In conclusion, CSCRSites is the first deep learning-based method for identifying cancer-specific RBP binding sites, which could contribute to the function analysis of cancer-associated circRNAs.

## 2. Results

In this section, in order to evaluate the performance of CSCRSites, it was compared with conventional machine learning classifiers and some existing representative deep learning-based methods for detecting RBP binding sites using the benchmark dataset CSCRBS (cancer-specific circRNA-RBP Binding sites).

### 2.1. Implementation of the Parameterized CSCRSites

CSCRSites was implemented in Python 3.7 using the Keras 2.2.4 library. Since the hyper-parameters of a deep learning model have a significant impact on its performance, we studied the different combinations of model settings and selected the model parameters with the best performance. After testing different kernel numbers in the range 128–1024, and various kernel sizes in the range from 8 to 50, we selected 1024 kernels with 8, 20, and 38 kernel sizes, respectively. In particular, test results show that deploying more convolutional kernels was always beneficial while the computation load is also increasing. Interestingly, in the max-pooling layer, the simple global pooling was superior than the local pooling strategy in each kernel.

In the training phase, the cross-entropy was used as the loss function, and the standard error back-propagation algorithm and Adam [34] method were adopted during the model training with the batch size of 512. Passing all training samples through the model and completing a back-propagation process once is an epoch. We trained our model till convergence and validated it after each epoch. In our tests, we found that 50 epochs were usually enough. Finally, the best model parameters were obtained according to the accuracy on the validation dataset using an early stopping strategy.

### 2.2. Performance of Different Combinations of CSCRSites Settings

**Figure 1:**
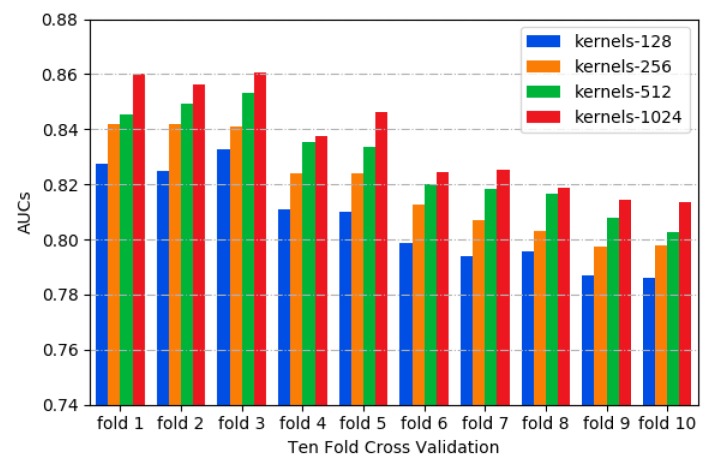
The distribution of AUCs across various kernels.

In this study, the area under the receiver operating characteristics curve (ROC_AUC) was used as a metric for model evaluation and comparison [35]. Different kernels can capture the variant features of sequences, such as motif variants in the motif discovery task. As shown in Figure 1, the 1024 kernels have the higher AUC values by testing different kernel numbers with 10-fold cross validation [36], indicating that more convolution kernels improve the performance of our model. However, the performance improvement of the model seems to be close to saturation when more than 1024 kernels were employed, while the computation load was also increasing. Thus, 1024 kernels were adopted in CSCRSites.

**Figure 2:**
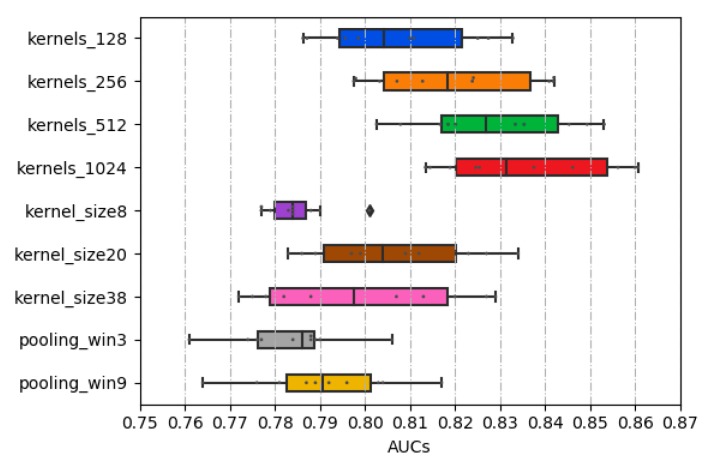
The distribution of AUCs across various parameters and structures.

As determined in Section 2.1, the kernel sizes of 8, 20, and 38 were employed through the concatenate strategy. Among them, the kernel size of 20 achieves the highest AUC value, as shown in Figure 2. Thus, in our model, the larger the convolution kernel size does not mean the better.

In general, local max-pooling is a common strategy in the deep learning-based model [26,27,32]. Global max-pooling was adopted in this study because the higher AUC values are obtained this way. As shown in Figure 2, the max-pooling sizes as Zeng’s method described [29] were experimented, and the result shows that the local max-pooling strategy achieves the worst performance.

### 2.3. Comparing CSCRSites with Conventional Machine Learning Methods

To utilize the sequences information in the conventional machine learning models, such as MLP (multilayer perception), SVM (support vector machine), and RF (random forest), k-mer compositional features are usually employed to encode RNA sequences as the inputs of models, in which each feature represents the normalized frequency of the corresponding k-mer appearing in an RNA sequence [37,38,39,40]. Here, the above-mentioned machine learning methods were implemented by adopting normalized 3-mer frequency representation of circRNA sequence fragments; it was a 4 × 4 × 4 or 64-dimensional vector, which was tested for the best performance. They were compared with the CSCRSites on the benchmark dataset CSCRBS. 

**Figure 3:**
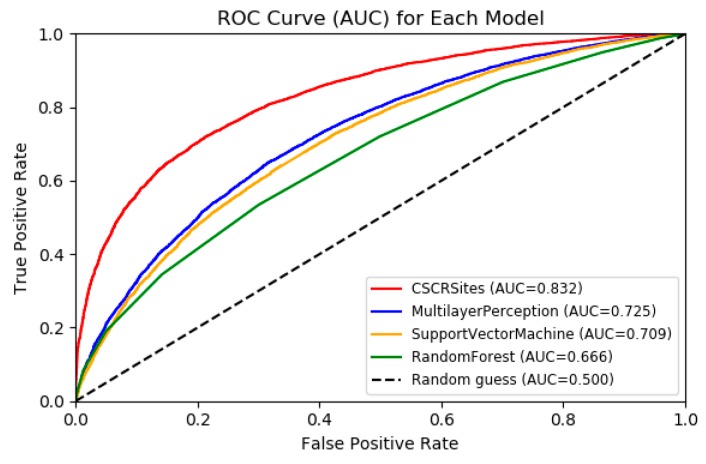
Receiver operating characteristics (ROC) curves to show the superior performance of CSCRSites (cancer-specific circRNA–RBP binding sites) over multilayer perception (MLP), support vector machine (SVM), and random forest (RF) on the test dataset. RBP, RNA binding protein.

As shown in Figure 3, CSCRSites obviously outperforms MLP, SVM, and RF according to the ROC curves; the AUC of CSCRSites is 0.8326, which is nearly 10% higher than that of the MLP (0.7249), which is the best performance in the conventional machine learning-based method, indicating the advantages of our model against conventional learning methods on the benchmark dataset CSCRBS.

### 2.4. Comparing CSCRSites with Existing Deep Learning Methods

**Figure 4:**
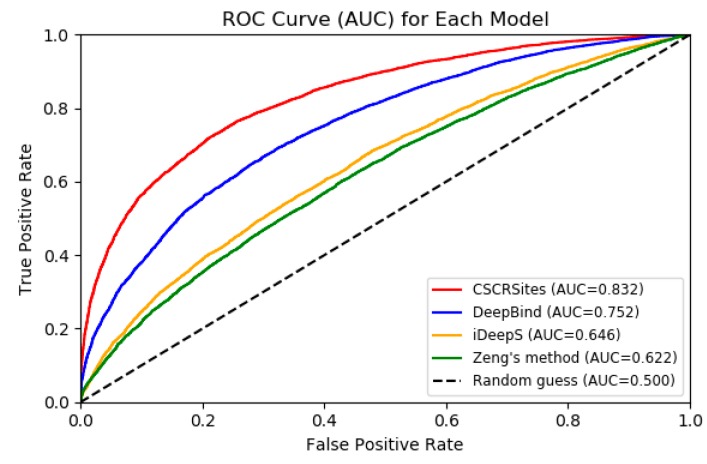
ROC curves to show the superior performance of CSCRSites over other deep learning-based methods on the test dataset.

In order to further verify the performance of CSCRSites, we compared it with some existing representative deep learning-based methods on dataset CSCRBS with the same evaluation criteria including accuracy (Acc.), precision (Prec.), and AUC. DeepBind [27] is the first deep learning based method for predicting the sequence specificities of DNA- and RNA-binding proteins. Zeng et al. [29] successfully apply the convolutional neural network architectures for predicting DNA-protein binding. iDeepS [32] was also developed to predict RBP binding sites on RNAs and have better performance than other existing methods, such as DeeperBind [28] and GraphProt [33]. Thus, we compared CSCRSites with DeepBind, Zeng’s method, and iDeepS on the dataset CSCRBS. The result is shown in Table 1 and Figure 4.

As shown in Table 1 and Figure 4, all methods have higher AUCs than a random guess (0.5). CSCRSites shows the best results, indicating its superior ability in predicting cancer-specific RBP binding sites on circRNAs. Specifically, CSCRSites achieves the highest AUC of 0.832. The Acc. and Prec. of CSCRSites are also higher than those of iDeepS, Zeng’s method, and DeepBind. In addition, the reason for iDeepS’ poor performance may be the lack of circRNA secondary structure information. These experimental results demonstrate that CSCRSites has superior ability in predicting cancer-specific RBP binding sites.

### 2.5. Performance of CSCRSites in Motif Discovery

As described in Section 4.4, the motifs learnt by CSCRSites were compared with the existing motifs using TOMTOM with an E value ≤0.05. All Species [41] was selected as the alignment database, as it is an RNA-binding motif database having 244 motifs between 7 and 8 in length, in which 102 motifs are *Homo sapiens* RNA-binding motifs. CSCRSites can learn the motifs of different lengths of 8, 20, and 38. Considering the length of motifs in the RNA-binding motifs database, the motifs with the length of 8 learnt by all kernels were aligned with the *Homo sapiens* RNA-binding motif database, and 65 motifs were significantly matched with 29 known motifs involving 23 genes. Some of the alignment results are shown in Table 2.

**Figure 5:**
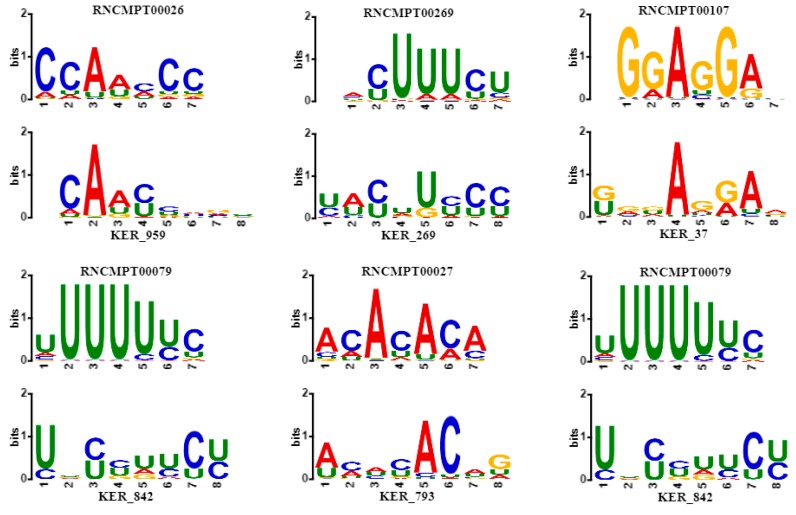
Some sequence logos of matched motifs whose associated genes are involved in human cancer. For each plot, the motifs learnt by CSCRSites (bottom) is aligned with the known motif (top) from *Homo* *sapiens* database by TOMTOM. The gene name associated with the known motif is shown in Table 2.

According to records in the database DisGeNET [42], some associated genes in Table 2 are encoding RNA-binding proteins that affect human diseases, especially human cancer. As shown in Figure 5, some sequence logos of matched motifs are associated with genes involving human cancer. For instance, HNRNPK overexpression is related to tumorigenesis in several cancers [43], whose binding motif RNCMPT00026 is matched with KER_959 learnt by CSCRSites. Similarly, in cutaneous melanoma, brain metastasis is predetermined by CD44 splicing variant 6 (CD44v6), whose expression correlates with PTBP1 and U2AF2 splicing factors, and especially, PTBP1 knockdown significantly decreases CD44v6 expression in advanced melanomas [44]; their binding motifs match to KER_269 and KER_842, respectively. TIA1, whose binding motif matched with KER_842, has an isoform expression that is measured in colorectal cancers [45]. RNA-binding protein HNRNPL has been previously shown to associate with tumorigenesis in liver and lung cancer [46], and results show that KER_793 matches with its binding motif RNCMPT00027. Splicing factor SRSF1 is an associated gene binding motif that matches with KER_37, which is upregulated in human breast tumors, and its overexpression promotes transformation of mammary cells [47].

## 3. Discussion

The experimental results show that CSCRSites, as the first model to predict cancer-specific RBP binding sites on circRNAs, is an effective computation method that outperforms the other methods for identifying the cancer-specific RBP binding sites on circRNAs. The application, merit, and demerits etc. of comparative methods are listed in Table 3. The better performance of CSCRSites is mainly attributed to the following aspects. Firstly, the application of a CNN provides the basic guarantee for the effectiveness of CSCRSites. A CNN can automatically obtain high-level features from nucleotide sequences and succeed in identifying RBP binding sites. Secondly, the reliable benchmark datasets of cancer-specific RBP binding sites on circRNAs are constructed while the circRNAs sequences are encoded to one-hot vectors that are order-preserving, which is more suitable for the motif discovery task compared with traditional k-mer feature extraction. Furthermore, employing multiple convolutional neural networks enhances the performance of our model, and is beneficial to various motif discovery tasks. Hence, CSCRSites is a flexible method of identifying cancer-specific RBP binding sites on circRNAs. 

Recent studies have reported that circRNAs could play their regulatory functions via sponging RBPs, therefore, it is necessary to understand the interaction mechanisms of circRNAs and RBPs, especially in human cancers. We trained the CSCRSites model to identify whether a given circRNA fragment was a cancer-specific RBP binding sites by using abundant binding sites data. Moreover, our model can detect the various length binding site motifs on circRNAs and provide reference for further researching on circRNA regulatory functions.

Despite the effectiveness of the CSCRSites model, it should be noted that CSCRSites still has some limitations. The model requires fixed-length binding sites as input data, as the binding sites are extended to 100 nt by centering at the point called for each peak, which may cause possible bias by abandoning length information. In future works, we plan to solve the problem of variable length input, try to collect more binding sites data, and integrate more circRNA information to improve the prediction accuracy of the model. Finally, we will develop a web tool for identifying the cancer-specific RBP binding sites.

## 4. Materials and Methods

**Figure 6:**
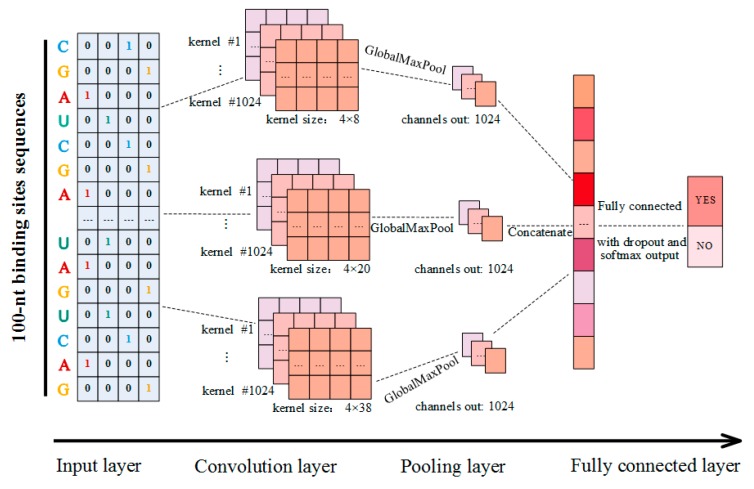
Schematic diagram of CSCRSites model construction.

In this study, CSCRSites, a method based on the CNN architecture, was constructed to identify the cancer-specific RBP binding sites on circRNAs. As shown in Figure 6, given a sequence fragment on circRNAs, and after converting it into a vector as the input, CSCRSites identifies whether the fragment is a cancer-specific RBP binding site. For this purpose, the cancer-specific RBP binding sites are collected from CSCD, which form the dataset for training and testing CSCRSites, respectively.

### 4.1. Datasets

In order to build the CSCRSites model for the RBP binding site prediction, we constructed a dataset of cancer-specific RBP binding sites on circRNAs. The RBP binding site information was extracted from the cancer-specific circRNAs database (CSCD, http://gb.whu.edu.cn/CSCD). It contains 15,719,824 RBP binding sites in cancer-specific circRNAs, 66,182,210 in normal circRNAs, and 22,025,003 in common circRNAs [15]. In the first place, cancer-specific RBP binding sites on circRNAs were downloaded from CSCD and treated as positive samples. Here, to obtain the determined RBP binding sites, 3026 cancer-specific circRNAs recorded by circBase [12] were selected, and 486,060 RBP binding sites were preserved after removing the redundant sites. Secondly, note that our model identifies the binding sites only based on circRNAs sequences. For the shorter sequences whose lengths were less than 50 nt, the features for distinguishing sequences were difficult to extract, and thus the sequences with lengths more than 50 nt and less than 100 nt were retained. In fact, for the longer sequences, nearly 80% of the binding sites were in this range. Finally, the sequences were extended to 100 nt by centering at the point called for each peak, and 43,118 cancer-specific RBP binding sites on circRNAs were obtained. The negative samples were obtained by shuffling positive sequences with matching dinucleotide composition [29]. The same strategy for creating negative samples is used in DeepBind [27]. The shuffling was implemented using the ‘fasta-dinucleotide-shuffle’ package in MEME [48]. The whole dataset was randomly divided into training and testing sets in the ratio of 4:1. The dataset was named CSCRBS.

In addition, the RBP binding sites sequences were collected according to the human GRCH37 genome from the UCSC Genome at https://genome.ucsc.edu.

### 4.2. Sequence Encoding

On the numerical descriptor of the sequences, k-mer compositional features were widely used, in which each sequence was encoded using a 4^K^-dimensional vector, and each feature represents the normalized frequency of the corresponding k-mer appearing in an RNA sequence [37]. This type of feature has difficultly in capturing the sequence order information, especially in the task of detecting motifs.

In this study, each sequence of the RBP binding sites on circRNAs was denoted as a numerical sparse matrix, and is an order-preserving transformation. Suppose that a circRNA sequence fragment $S=‘s_{1}s_{2}s_{3}\cdots s_{L}’$, where L is the length of a circRNA sequence fragment, $s_{j}\in \left\{A,U,C,G\right\},j=1,2,3,\cdots ,L$, which are denoted as one-hot vectors [1,0,0,0], [0,1,0,0], [0,0,1,0], and [0,0,0,1], respectively. Then, the circRNA sequence fragment S can be represented as follows:

(1) $$M={\left(m_{i,j}\right)}_{4\times L},\;m_{i,j}=\{\begin{array}{c} 1,s_{j}=h\left(i\right)\\ 0,\;otherwise \end{array}\;i=1,2,3,4;\;j=1,2,3,\cdots L,\;h\left(i\right)=\left[A,U,C,G\right].$$

Finally, through the one-hot encoding, the circRNA sequence fragment S is characterized by a 4 × L numerical sparse matrix M.

In our study, all RBP binding sites on circRNAs were encoded to a corresponding 4×L matrix M as the input of the model. This encoding strategy can not only obtain low dimension data, but also maximize the retention of the sequence original information, and thus it is conducive to extract features using the deep learning-based method. Especially, it is beneficial to the task for motif discovery.

### 4.3. Model Construction

A CNN is a neural network that uses convolution in place of general matrix multiplication in at least one of its layers [49]. In recent years, the CNN was applied to model sentences [50] to detect features of the raw input data by the convolution operation. By this inspiration, a CNN was applied to extract the features of genomic sequences, and the kernels or filters in a CNN were used for motif discovery [27,51]. In this study, we constructed our parameterized model based on a CNN to learn the sequence features of cancer-specific RBP binding sites on circRNAs, and to further identify whether a new circRNA sequence fragment was a cancer-specific RBP binding site.

The structure of our model is shown in Figure 6; it is a slight variant of the TextCNN [52], and contains an input layer, a convolution layer, a pooling layer, and a fully connected layer. Suppose that $x_{i}$ is the 4-dimensional binary vector corresponding to the i-th nucleotide $S_{i}$ on a circRNA sequence fragment as described in Section 4.2, and a circRNA sequence fragment of length L is expressed as follows:

(2) $$M=x_{1}⊕x_{2}⊕\cdots ⊕x_{L}$$

where ⊕ is the concatenation operator.

The convolution layer is used to learn the features of different levels. Considering $c_{i}$ is a new feature generated by the convolution operation on matrix *M*, it can be described as follows:

(3) $$c_{i}=f\left(w*x_{i:i+h-1}+b\right)$$

where f(x) denotes a non-linear activation function, such as the rectified linear unit (ReLU), operator ∗ represents the convolution operation, b is a bias term, w is the weight matrix of the convolution kernel, and $x_{i:i+h-1}$ refers to the concatenation of nucleotide $x_{i},x_{i+1},\;\cdots ,\;x_{i+h-1}$. Here, h is the kernel size (the width of matrix w). The kernel is applied to different fragments of a sequence with a fixed window $h\;\{x_{1:h},x_{2:h+1},\;\cdots ,\;x_{L-h+1:L}\}$ in turn, then produces a feature map:

(4) $$c=\left[c_{1},c_{2},\cdots ,c_{L-h+1}\right].$$

In this study, our model employs multiple convolution layers with different kernel sizes to extract diverse features. In addition, multiple kernels were employed to extract the features in each convolution layer. As shown in Figure 6, three different kernel sizes were applied to detect diverse features of circRNA sequence fragments, while 1024 kernels were employed with each kernel size by testing for the best performance.

The pooling layer is used to compress data and reduce over-fitting. To obtain the most important feature on a feature map c, a max-pooling operation is applied to each feature map [53]:

(5) $$\hat{c}=\max\left\{c\right\}.$$

That is, the maximum value of a feature map denotes the feature of the corresponding kernel, in this study, as shown in Equation (5), a simple global max-pooling strategy is adopted. Then, outputs are concatenated together to form a single feature vector and are fed into a fully connected layer. 

The fully connected layer with the non-linear softmax activation function is used to classify the extracted features, and the outputs are the probability distribution over labels. Deserving to be mentioned, to avoid over-fitting, a dropout layer was implemented before this layer. The CSCRSites can be summarized as follows:

(6) $$Out=f^{Fc\_softmax}f^{Concatenate}f^{GlobalMaxPool}f^{Conv2\_ReLU}\left(M\right)$$

where the M is the input sparse matrix in Section 4.2, and the Out is the probability whether a sequence fragment is a cancer-specific RBP binding site.

### 4.4. Motifs Discovery

As described in the previous study [54,55], the convolution layers of CSCRSites are akin to motif scanners. As described in Section 4.3, given an input sequence, a feature map c could be produced for each kernel via the convolution layer with the non-linear ReLU function. Here, the position of the maximum value $c_{i}$ in the feature map c is regarded as the start position of a motif. The motif position information matrix is calculated using the Python API (keras.K.function), which is defined as follows:

(7) f_matrix=K.function(M,[K.argmax(c),K.max(c)])

where the matrix M is the model input, c is the feature map in each kernel, that is, the output of the convolution layer. The function K.argmax and K.max are keras backend functions returned the position and value of maximum value $c_{i}$, respectively. Finally, matrix f_matrix is converted to motifs with the MEME motif format, and afterwards, compared with the existing motifs by submitting to the TOMTOM [56] webserver.

## 5. Conclusions

In this study, we have developed CSCRSites as the first model to identify cancer-specific RBP binding sites on circRNAs based on convolutional neural network architecture. To obtain the initial sequence information for the motif discovery task, circRNA sequences were represented by one-hot encoding, then relevant features were automatically extracted from nucleotide sequences by concatenating multiple convolutional neural networks. The achieved high-level features were fed to a fully connected layer with a non-linear softmax activation function for the classification. In addition, CSCRSites also can be used as a motif discovery tool to discover three kinds of different motifs using the features learnt by CNN layers. The goal of using multiple convolutional neural networks is to obtain various high-level features for the softmax classifier and identify the different length motifs in the motif discovery task. Compared with existing deep learning-based models and the conventional machine learning methods, our model has an advantage in identifying cancer-specific RBP binding sites. In addition, it can be extended to identify the tissue-specific and cell-specific circRNA expression patterns. We hope that our method presented in this study can contribute to further models and understanding of the functions of circRNAs.

## Figures and Tables

**Table 1:** CSCRSites outperforms other deep learning-based models on the test dataset. Accuracy, Acc.; precision, Prec.

	Acc.	Prec.	AUC
CSCRSites	0.74	0.76	0.83
DeepBind	0.68	0.68	0.75
iDeepS	0.61	0.64	0.65
Zeng’s method	0.59	0.59	0.62

**Table 2:** Some motifs learnt by CSCRSites are aligned with the known motifs and the associated genes.

Associated Genes	Known Motifs ID	Known Sequence	Learnt Motifs ID	Learnt Sequence	Overlap	E-Value
DAZAP1	RNCMPT00013	UAGGUAG	KER_29	UAGGUAGG	7	0.0031
FMR1	RNCMPT00016	GGACAAG	KER_632	GGCACAGG	7	0.0290
HNRNPK	RNCMPT00026	CCAACCC	KER_959	CAACCAGU	6	0.0429
HNRNPL	RNCMPT00027	ACACACA	KER_793	ACACACAG	7	0.0019
HNRPLL	RNCMPT00178	ACACACA	KER_793	ACACACAG	7	0.0030
HuR	RNCMPT00032	UUAUUUU	KER_78	UUUAUUUU	7	0.0054
	RNCMPT00112	UUUGUUU	KER_900	UUUCUUUC	7	0.0098
	RNCMPT00117	UUUGUUU	KER_900	UUUCUUUC	7	0.0070
	RNCMPT00136	UUGGUUU	KER_395	AUUGAUUU	7	0.0202
IGF2BP2	RNCMPT00033	ACAAACA	KER_512	AAACACAG	7	0.0401
IGF2BP3	RNCMPT00172	ACAAACA	KER_793	ACACACAG	7	0.0110
KHDRBS1	RNCMPT00169	AUAAAAG	KER_837	UAUUAAAG	7	0.0254
MATR3	RNCMPT00037	AAUCUUG	KER_801	GAAUCUUG	7	0.0021
PABPC5	RNCMPT00171	AGAAAAU	KER_113	AGAAAGUG	7	0.0060
PABPN1	RNCMPT00157	AGAAGAC	KER_183	AGAAAACA	7	0.0109
PCBP1	RNCMPT00186	CCUUUCC	KER_577	CCUUCCCU	7	0.0055
PCBP2	RNCMPT00044	CCUUCCC	KER_577	CCUUCCCU	7	0.0021
PTBP1	RNCMPT00268	CUUUUCU	KER_366	UUUUCUUU	6	0.0208
	RNCMPT00269	ACUUUCU	KER_269	UACUUCCC	7	0.0051
RBM46	RNCMPT00054	AAUCAAU	KER_153	GAAUCAAU	7	0.0208
SAMD4A	RNCMPT00063	GCUGGAC	KER_608	UGCUGGCC	7	0.0347
SNRNP70	RNCMPT00070	GAUCAAG	KER_197	GAAUCAAG	7	0.0065
SRSF1	RNCMPT00107	GGAGGAA	KER_37	GGGAGGAA	7	0.0391
SRSF10	RNCMPT00019	AGAGAAA	KER_824	AGAGAAAA	7	0.0373
	RNCMPT00089	AGAGAAA	KER_824	AGAGAAAA	7	0.0299
TIA1	RNCMPT00165	UUUUUUC	KER_842	UUCCUUCU	7	0.0122
U2AF2	RNCMPT00079	UUUUUUC	KER_842	UUCCUUCU	7	0.0036
ZC3H14	RNCMPT00086	UUUGUUU	KER_900	UUUCUUUC	7	0.0111

**Table 3:** Application, merit, and demerit of comparative methods.

Methods	Application	Motifs	Merit	Demerit
CSCRSites	circRNA binding sites	YES	Discovery of various length motifs High prediction accuracy	The rate of convergence is relatively slow
DeepBind	DNA/RNA binding sites	YES	Scales well to ChIP-seq and HT-SELEX data sets	Low prediction accuracy on circRNA data sets
iDeepS	RNA binding sites	YES	Integrates RNA secondary structure	Predict binding targets for specific RBP
Zeng’s method	DNA binding sites	YES	Motif occupancy task	Motif length is fixed
